# A Piezoelectric MEMS Microgripper for Arbitrary XY Trajectory

**DOI:** 10.3390/mi13111888

**Published:** 2022-11-01

**Authors:** Fabio Botta

**Affiliations:** Depatment of Industrial, Electronic and Mechanical Engineering, Roma Tre University, Via Vito Volterra 62, 00146 Roma, Italy; fabio.botta@uniroma3.it; Tel.: +39-06-5733-3491

**Keywords:** MEMS, piezoelectric, path generator

## Abstract

In this paper, a piezoelectric microgripper for arbitrary 2D trajectory is proposed. The desired trajectory of the specimen under consideration was obtained by the deformability of a structure consisting of 16 straight beams and 12 C-structures. The mechanical action that deforms the structure was obtained by an electrical voltage supplied to piezoelectric plates. In order to verify the proposed model a FEM software (COMSOL) was used and some of the most commonly used trajectories for medical applications, micropositioning, micro-object manipulation, etc., were examined. The results showed that the proposed microgripper was capable of generating any parametrizable trajectory. Parametric studies were also carried out by examining the most relevant parameters highlighting their influence on specimen trajectories.

## 1. Introduction

Lately MEMS have undergone considerable development because of their applications in many fields: micropositioning [1], micro-object manipulation [2,3,4], Lab-on-Chip [5], sensors [6], and energy harvesting [7]. In particular, MEMS capable of making a body perform particular 2D trajectories have been used in: biological micro-/nanomanipulation [8,9], scanning probe microscopy based nanoimaging [10,11], micro-opto-electromechanical systems [12], etc. Rather complex mechanisms are typically used in this regard [13,14,15] or systems using two piezoelectric translator actuators (PEAs) arranged orthogonally in the plane of the desired trajectory [16,17,18]. In fact, piezoelectric materials due to their high response speed, wide frequency bandwidth, etc., are widely used in precision engineering, soft materials analysis and characterization [19] harvesting, vibration control [20,21,22,23], etc. However, PEAs typically cannot produce large displacements (a 10 mm long PZT generally has a stroke of 10 microns [24]), and this results in a frequent need of displacement amplification systems such as flexure hinge-based compliant, Scott Russell [25], Z-shaped [26], bridge-type [16], and rhombic mechanisms, the displacements are amplified up to tens of micrometers [27]. In recent years, these have been used less and less because the use of such complex systems worsens the dynamic and static characteristics of the system by reducing its structural stiffness and intrinsic resonant frequency [27]. In contrast, there are a few studies that directly use the piezoelectric effect on the structure to achieve the desired trajectory [28,29].

In this paper, a new device is proposed that uses a different approach with respect to those described previously. The structure is symmetrical with respect to the *x*-axis, and on the same axis, the element to be moved is placed. Unlike devices using PEAs, in this case, the actions of piezoelectrics are directly exploited: in fact, piezoelectric plates are used to deform the structure and through this deformation the displacement of the specimen is obtained. Such a structure is, basically, divided into two parts: *x*-displacement unit and *y*-displacement units. In the first part, the piezoelectric actions are symmetrical with respect to the *x*-axis so that the specimen can displace only along that axis, while in the second part they are asymmetrical so that they can displace only along the *y*-axis. By combining these two actions, any kind of 2D trajectory can be obtained.

The characteristics of the proposed mechanism are the simplicity of its construction (it does not need displacement amplification systems) and its versatility. In fact, in contrast to other mechanisms that directly use the piezoelectric effect on the structure, allowing one to realize only simple trajectories, in this case, all kinds of parametric trajectories can be realized, switching from one to another by simply changing the electrical voltage supplied to the piezoelectric plates. In addition, again by varying the voltage, the working range can be changed from a few microns to tens of microns. Such trajectories can be applied in many fields such as micromanipulation, medical treatments (such as the removal of calcifications and obstructions present on arterial walls), to investigate, at the atomic level, the physical properties of matters, etc. This system could, in the future, take advantage of piezoelectric nanogenerators [30,31] or other power systems [32] to build a self-powered device.

## 2. Analytical Model

**Figure 1:**
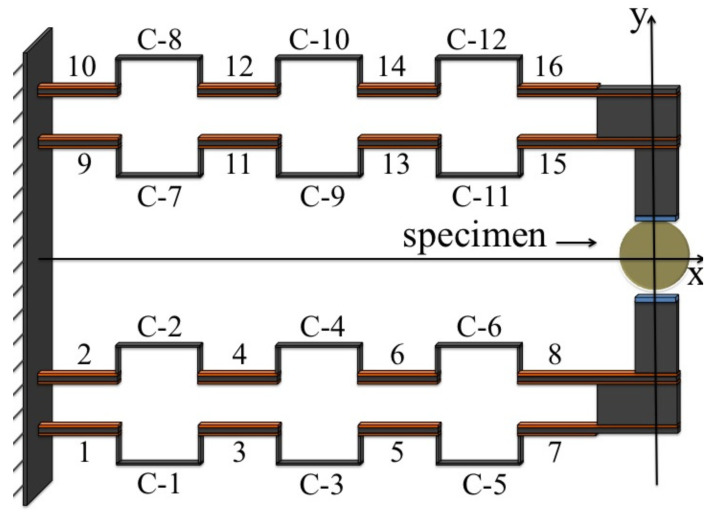
Geometry of the proposed piezoelectric-based MEMS device.

**Figure 2:**
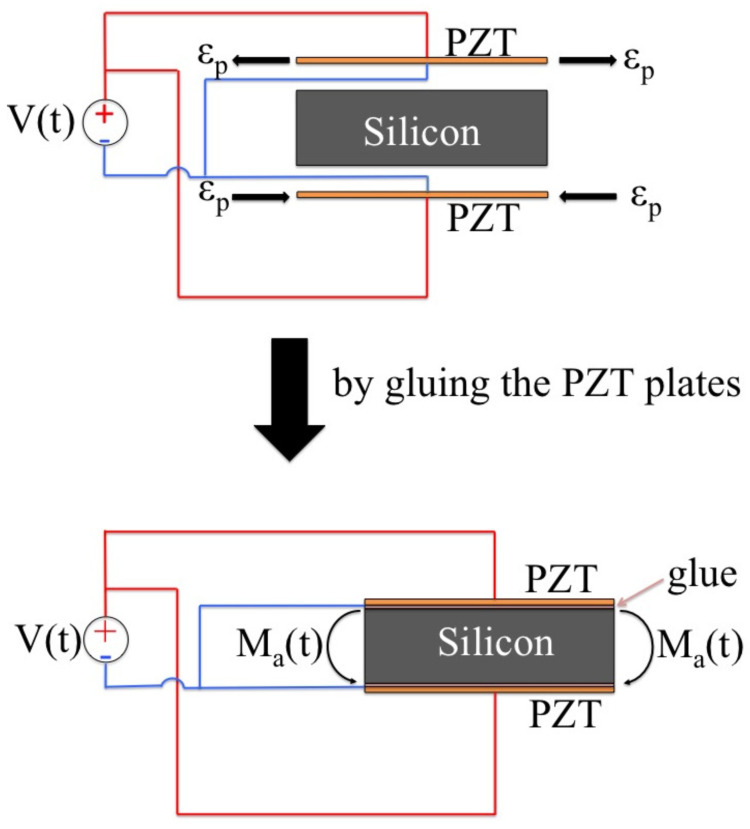
A schematic representation of the pin force model.

A schematic of the proposed model is shown in Figure 1:

The structure consists of 16 rectilinear beams connected to each other by 12 C-beams; to each rectilinear beam are symmetrically attached two piezoelectric plates (in orange in the figure). The displacement of the specimen is obtained by deforming the structure by means of the action of the piezoelectric plates. In fact, by supplying an electrical voltage to such plates, they will tend to deform (see Figure 2); by bonding them to the beam, this deformation will be partially limited and so they will apply a stress state to the beam. Several studies [33] have shown that this stress state is concentrated at the end of the plates, and the action on the beam can be represented, in essence, by two bending moments $M_{a}\left(t\right)$:

where (see [23,33,34]):

(1) $$M_{a}\left(t\right)=\frac{\psi }{6+\psi }E_{p}bh_{p}h_{M}\Lambda \left(t\right)$$

and

(2) $$\left\{\begin{array}{c} \psi =\frac{E_{M}h_{M}}{E_{p}h_{p}}\\ \Lambda \left(t\right)=\frac{d_{31}}{h_{p}}V\left(t\right) \end{array}\right.$$

**Figure 3:**
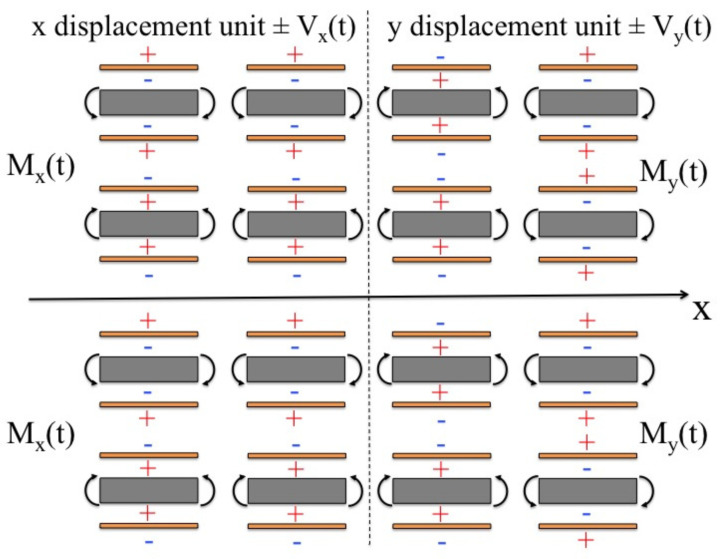
Example of symmetrical and antisymmetrical loads with respect to the *x*-axis. $M_{x}\left(t\right)$ and $M_{y}\left(t\right)$ denote, respectively, the bending moments which produce only *x*-axis and *y*-axis motion of the tip.

The purpose of C-beams is to reduce the axial stiffness of the entire structure by allowing appreciable displacements in that direction. The symmetry of the structure with respect to the *x*-axis, and the placement of the specimen on the same axis, allows the specimen to move only along this axis when the load applied to the structure is also symmetrical. On the other side, it can move only along the *y*-axis when that load is asymmetrical. By combining these two actions, the specimen can take any trajectory in the *x*–*y* plane. For this purpose, the system was divided into two parts: *x* displacement unit and *y* displacement unit (see Figure 3):

The distribution of electrical voltages was such that it provided only symmetrical loads in the first unit and only antisymmetrical loads in the second unit.

The voltage supplying the *x*-unit was denoted by $V_{x}\left(t\right)$, to which the applied moment $M_{x}\left(t\right)$ corresponds, and the voltage supplying the *y*-unit, to which the applied moment $M_{y}\left(t\right)$ corresponds, was denoted by $V_{y}\left(t\right)$. Denoting by u(t) and v(t) the displacements of the specimen along the *x*- and *y*-axes, the following could be written:

(3) $$\left\{\begin{array}{c} u\left(t\right)=B_{x}M_{x}\left(t\right)\\ v\left(t\right)=B_{y}M_{y}\left(t\right) \end{array}\right.$$

where the constants $B_{x}$ and $B_{y}$ depend on the configuration of the structure, boundary conditions, material properties, etc. Considering (1) and (2), (3) becomes:

(4) $$\left\{\begin{array}{c} u\left(t\right)=C_{x}V_{x}\left(t\right)\\ v\left(t\right)=C_{y}V_{y}\left(t\right) \end{array}\right.$$

with:

(5) $$\left\{\begin{array}{c} C_{x}=B_{x}\frac{\psi }{6+\psi }E_{p}bh_{M}d_{31}\\ C_{y}=B_{y}\frac{\psi }{6+\psi }E_{p}bh_{M}d_{31} \end{array}\right.$$

If $x_{p}\left(t\right)$ and $y_{p}\left(t\right)$ represent the parametric equations of the desired trajectory for the specimen, it suffices to pose:

(6) $$\left\{\begin{array}{c} u\left(t\right)=x_{p}\left(t\right)\\ v\left(t\right)=y_{p}\left(t\right) \end{array}\right.$$

which, with (4), becomes:

(7) $$\left\{\begin{array}{c} V_{x}\left(t\right)=\frac{1}{C_{x}}x_{p}\left(t\right)\\ V_{y}\left(t\right)=\frac{1}{C_{y}}y_{p}\left(t\right) \end{array}\right.$$

from which the tensions necessary to execute the desired trajectory can be derived. In this way any trajectory can be achieved.

## 3. Results and Discussion

A multiphysics FEM software tool (COMSOL) was utilized to verify the proposed microdevice. Typical MEMS material (silicon) was used for the structure while PZT-5A was chosen for the piezoelectric plates. The material properties are given in Table 1.

**Figure 4:**
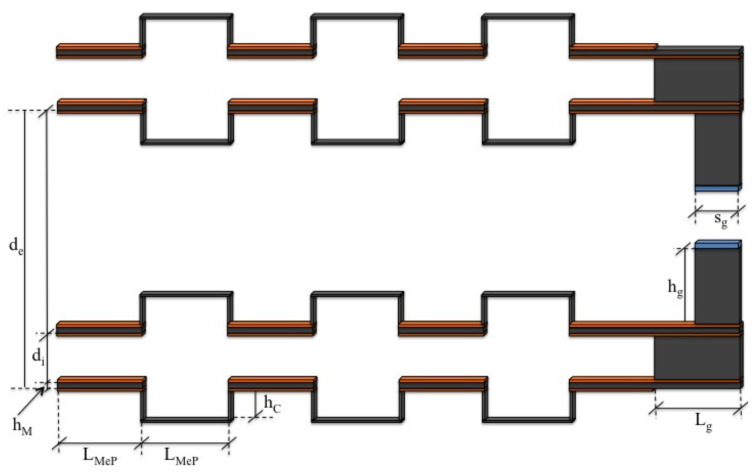
Relevant dimensions of the proposed piezoelectric-based MEMS device.

The details of the geometry and the values of the different quantities are reported in Figure 4 and Table 2:

To test the potential of the proposed model some of the most commonly used trajectories for micro-object manipulation, micropositioning, medical treatment (endoluminal treatment of obstructive lesions, microsurgical operations, arteries unclogging), etc., were examined. The list of trajectories and their electrical voltages used are shown in Table 3.

**Figure 5:**
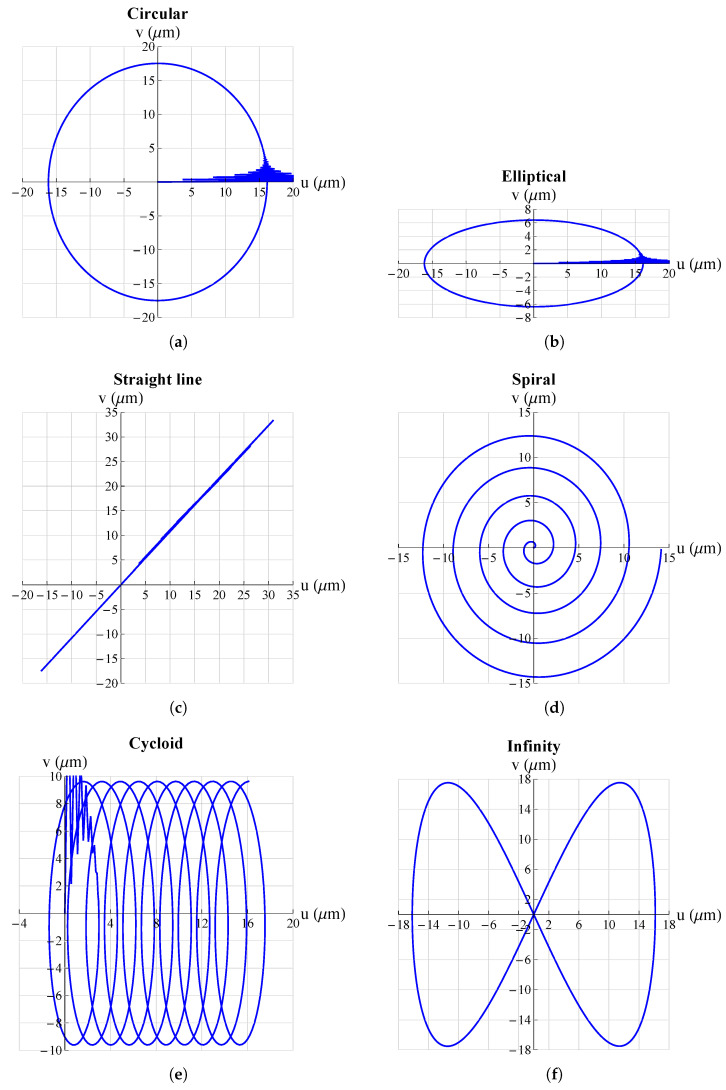
Some possible trajectories applicable, just to mention a few examples, in the medical field for atherectomy operations: (**a**) circular; (**b**) elliptical; (**c**) straight line; (**d**) spiral; (**e**) cycloid; (**f**) infinity. All graphs were obtained by means of FEM simulations.

**Figure 6:**
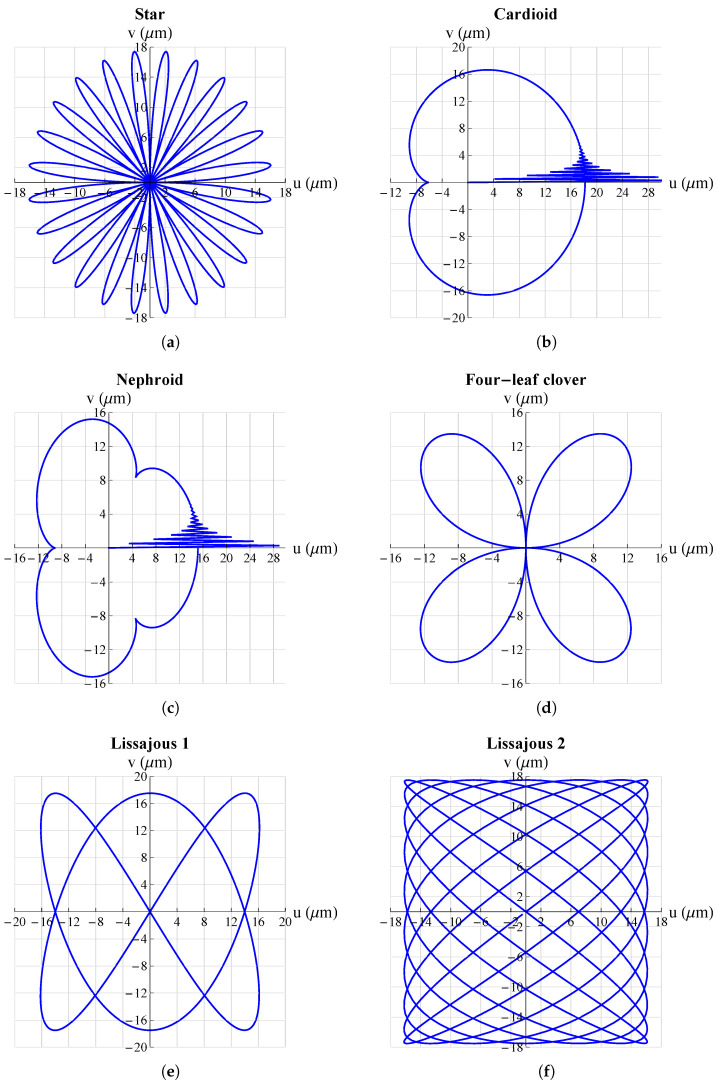
Some possible trajectories applicable, just to mention a few examples, for scanning methods in atomic force microscopes (AFM): (**a**) star; (**b**) cardioid; (**c**) nephroid; (**d**) four-leaf clover; (**e**) Lisaajous-1; (**f**) Lissajous 2. All graphs were obtained by means of FEM simulations.

The results are shown in Figure 5 and Figure 6. They show that the mechanism was able to follow all set trajectories. Only in some cases was there a small initial oscillation due to the fact that the mechanism always started from the origin and if the initial point of the trajectory was not at the origin there was an initial *“step”* that resulted in such oscillations. However, these were quickly damped and then the mechanism followed the set trajectory.

**Figure 7:**
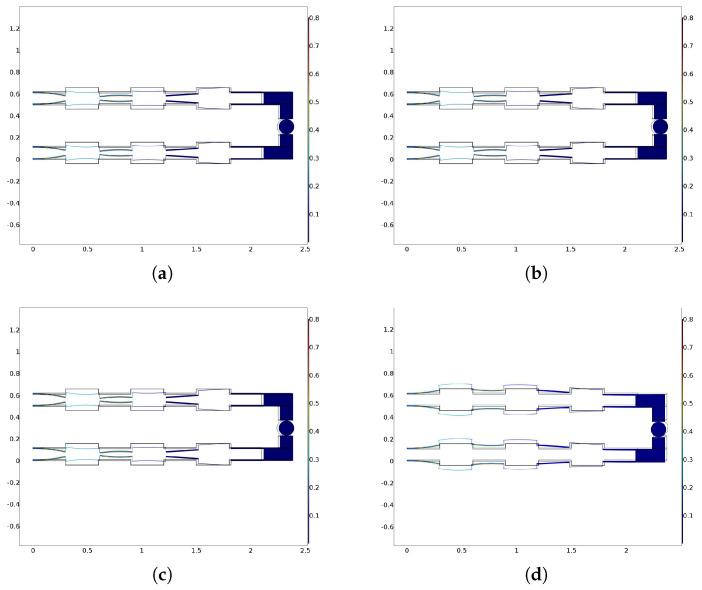
Von Mises stress for (**a**) circular, (**b**) elliptical, (**c**) cycloidal, and (**d**) Lissajous 1 trajectories. All graphs were obtained by means of FEM simulations. Stress scale is in GPa.

In order to verify that the stresses did not exceed the maximum allowable value, the Von Mises stress plots are shown for some trajectories in the most severe situations (Figure 7). It can be observed that the stresses never exceeded 2 GPa, well within the preyield stress.

**Figure 8:**
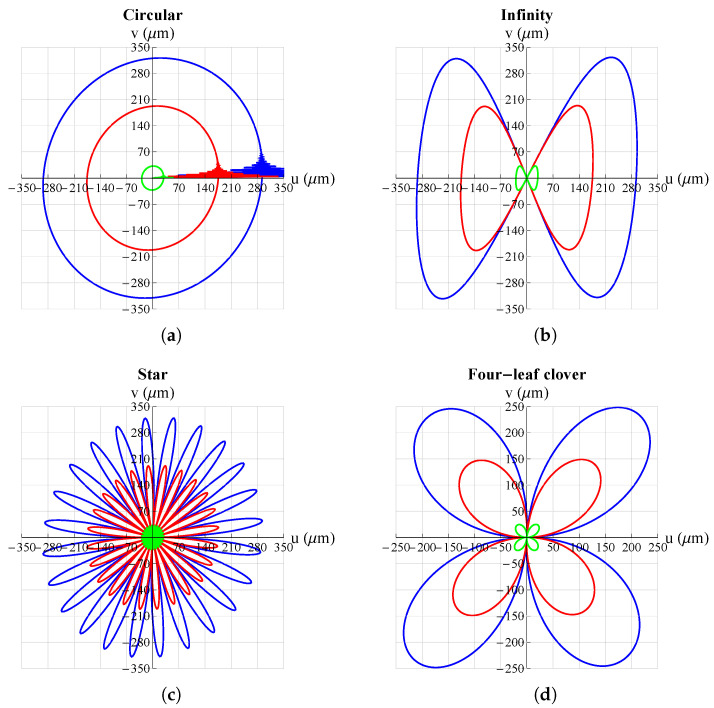

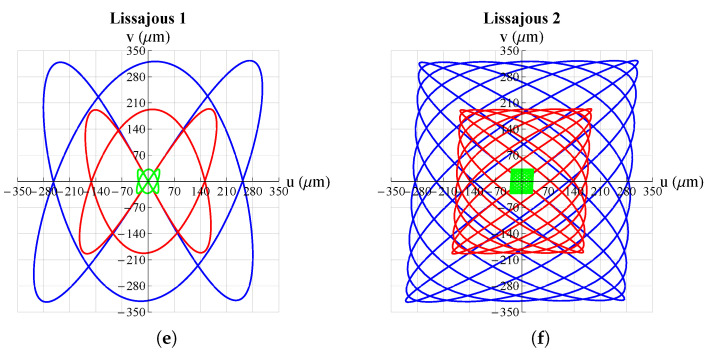
Effect of the electrical voltage supplied to piezoelectric plates on different trajectories. _______: $\{V_{x}$ = 50 V, $V_{y}$ = 20 V}; _______: $\{V_{x}$ = 30 V, $V_{y}$ = 12 V}; _______: $\{V_{x}$ = 5 V, $V_{y}$ = 2 V}: (**a**) circular; (**b**) infinity; (**c**) star; (**d**) four-leaf clover; (**e**) Lissajous 1; (**f**) Lissajous 2. All graphs were obtained by means of FEM simulations.

The working space could be changed, for each trajectory, simply by varying the amplitude of the electrical voltage supplied to the piezoelectric plates. Figure 8 shows some results in which the same type of trajectories were obtained with different voltages. It can be seen that the mechanism was able to go from a few microns to hundreds of microns.

**Figure 9:**
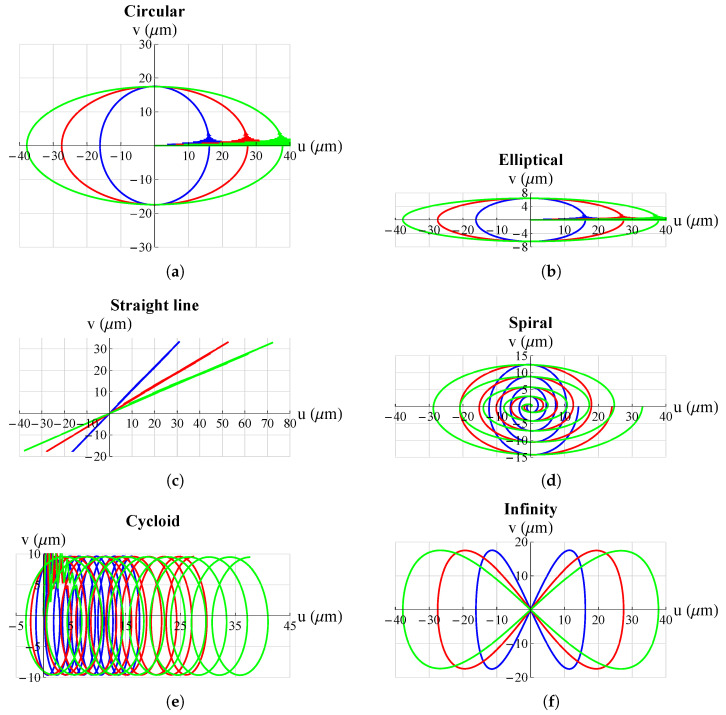
Effect of dimension $h_{C}$ on different trajectories. _______: $h_{C}=36$ μm, _______: $h_{C}=72$ μm, _______: $h_{C}=108$ μm: (**a**) circular; (**b**) elliptical; (**c**) straight line; (**d**) spiral; (**e**) cycloid; (**f**) infinity. All graphs were obtained by means of FEM simulations.

**Figure 10:**
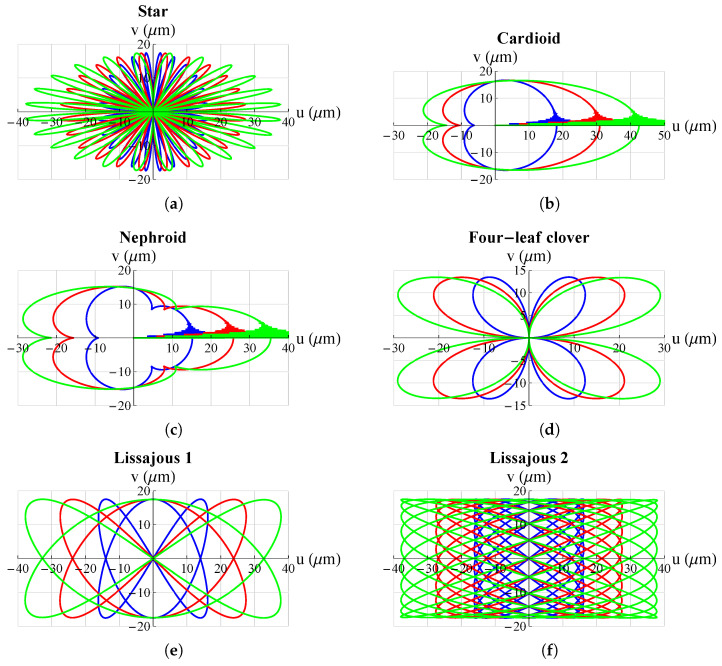
Effect of dimension $h_{C}$ on different trajectories. _______: $h_{C}=36$ μm, _______: $h_{C}=72$ μm, _______: $h_{C}=108$ μm: (**a**) star; (**b**) cardioid; (**c**) nephroid; (**d**) four-leaf clover; (**e**) Lisaajous-1; (**f**) Lissajous 2. All graphs were obtained by means of FEM simulations.

The effects of the geometrical dimensions on the amplitude of the trajectories were also investigated. They were different, in accordance with the type of parameter being considered. The first to be examined see Figure 9 and Figure 10 was the height of the connecting C-structure between the straight beams ($h_{C}$ in Figure 4).

**Figure 11:**
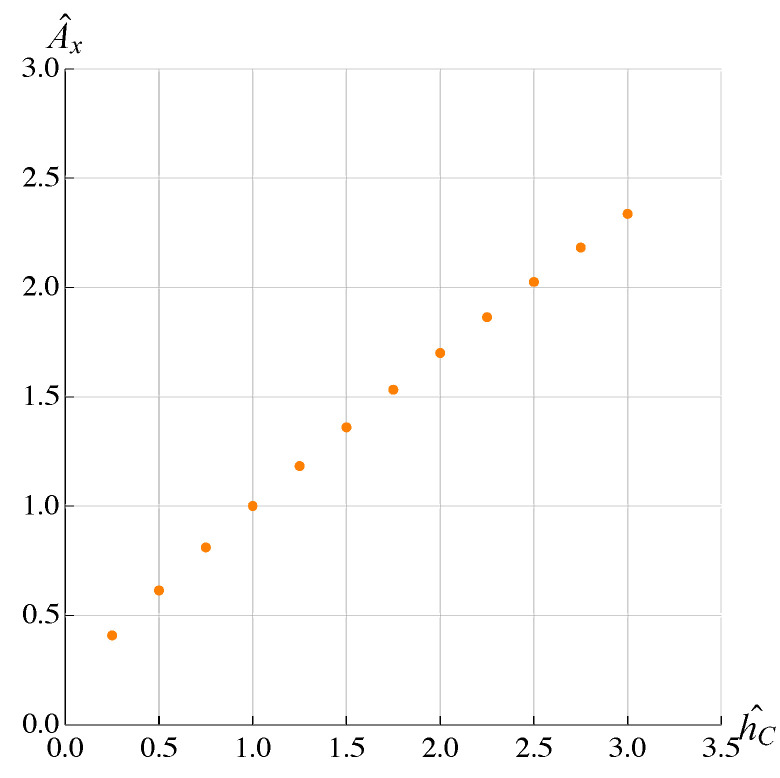
Effect of dimension $h_{C}$ on $\overset{\wedge }{A_{x}}$.

From the analysis of the figures, it can be seen that $h_{C}$ had an effect only on the excursion in the *x*-direction and not in the *y*-direction or, in other words, this parameter essentially affected only the stiffness of the structure in the *x*-direction. Moreover, these variations did not depend on the type of trajectory; the results are summarized in Figure 11:

where $\overset{\wedge }{h_{C}}$ is the dimensionless value of $h_{C}$ with respect to the chosen reference value present in Table 2 and $\overset{\wedge }{A_{x}}$ is the amplitude variation with respect to the reference amplitude. It can be observed that the variation is linear.

**Figure 12:**
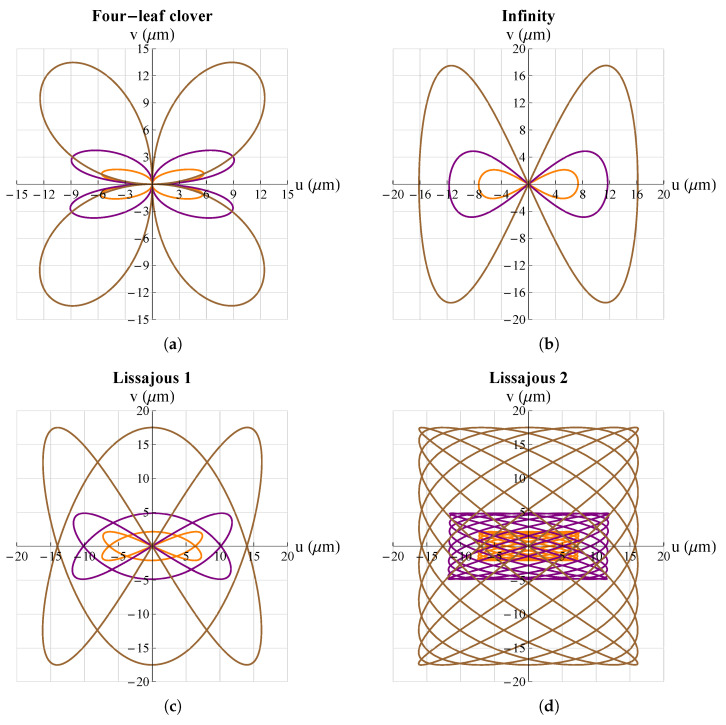
Effect of dimension $L_{MeP}$ on different trajectories. **_______**: $L_{MeP}=300$ μm, **_______**: $L_{MeP}=225$ μm, **_______**: $L_{MeP}=150$ μm: (**a**) four-leaf clover; (**b**) infinity; (**c**) Lissajous 1; (**d**) Lissajous 2. All graphs were obtained by means of FEM simulations.

The second parameter examined was $L_{MeP}$. The results are shown in Figure 12 (by way of illustration, not all cases are reported but only some simulations).

**Figure 13:**
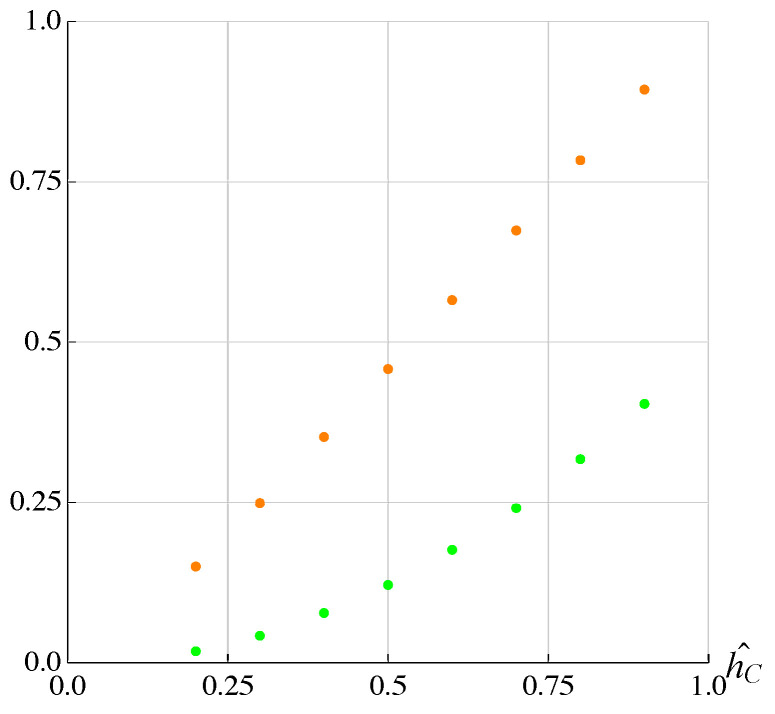
Effect of dimension $L_{MeP}$ on $\overset{\wedge }{A_{x}}$ (in orange) and $\overset{\wedge }{A_{y}}$ (in green).

In this case, it can be seen that the parameter impacted the amplitude of the working range on both the *x*- and *y*-axes; however, this effect was more pronounced on the *y*-axis than on the *x*-axis. In order to highlight these changes, a graph summarizing the results obtained are shown in Figure 13.

**Figure 14:**
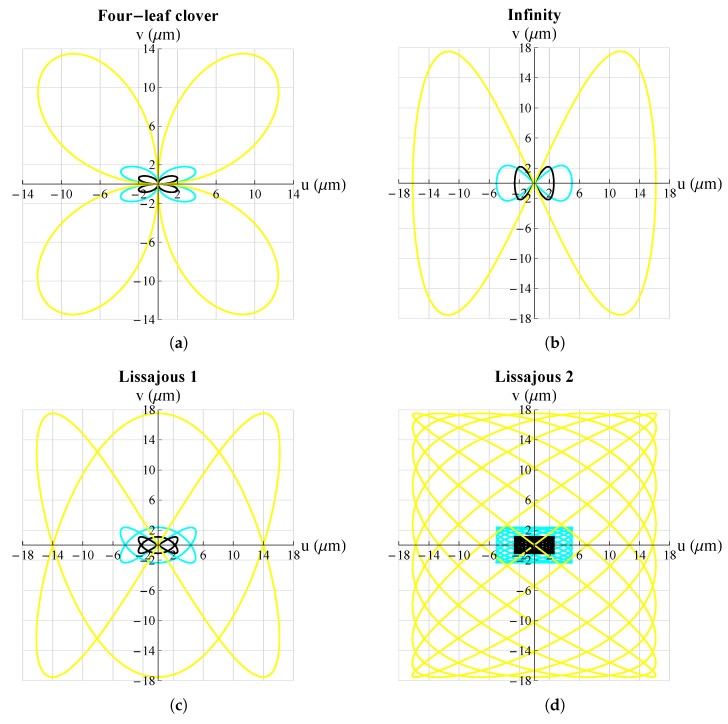
Effect of dimension $h_{M}$ on different trajectories. **_______**: $h_{M}=10$ μm, **_______**: $h_{M}=20$ μm, _______: $h_{M}=30$ μm: (**a**) four-leaf clover; (**b**) infinity; (**c**) Lissajous 1; (**d**) Lissajous 2. All graphs were obtained by means of FEM simulations.

Finally, the effect of the MEMS thickness $h_{M}$ was studied (Figure 14).

**Figure 15:**
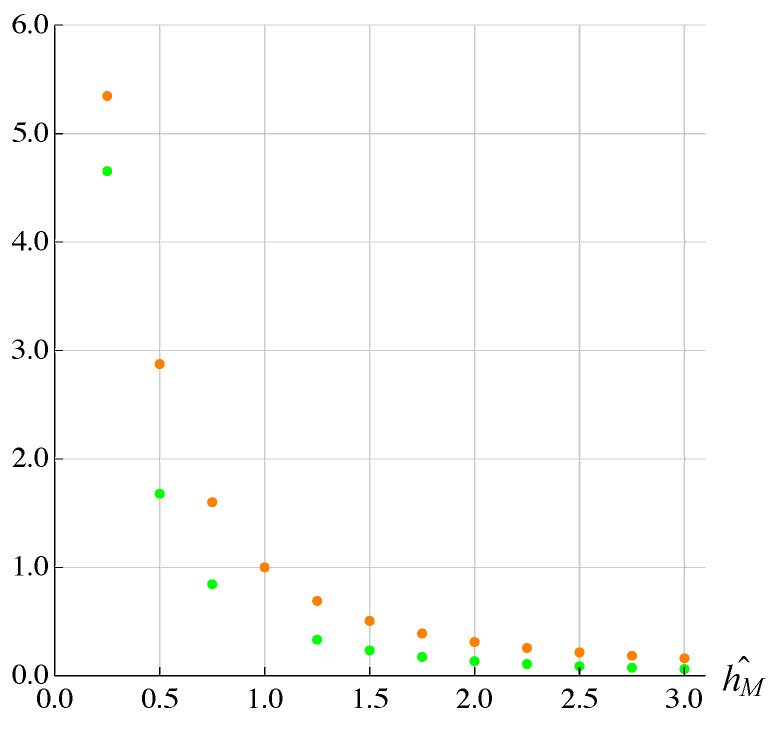
Effect of dimension $h_{M}$ on $\overset{\wedge }{A_{x}}$ (in orange) and $\overset{\wedge }{A_{y}}$ (in green).

From the figure it can be seen that $h_{M}$ also affected the working range on both axes but this time, the effect seemed essentially of the same type; in Figure 15 the results obtained for the cloverleaf trajectory are shown.

## 4. Conclusions

A new microgripper was proposed. The grasping and displacement of the specimen was accomplished by deforming a symmetrical structure through the action of piezoelectric plates. It was shown that the proposed system was capable of performing any type of plane parametric trajectory with a displacement head ranging from a few microns to hundreds of microns simply by acting on the voltage that was supplied to the piezoelectric plates. A parametric study was also conducted to highlight the effect of certain geometrical characteristics of the structure on the amplitude of the trajectories.

## Figures and Tables

**Table 1:** Materials properties.

Property	Silicon	PZT-5A	Unit
Density	2329	7750	kg/m^3^
Poisson’s ratio	0.28	–	–
Young’s modulus	170	–	GPa
$d_{31}=d_{32}$	–	−1.71	$10^{-10}$ C/N
$d_{33}$	–	3.74	$10^{-10}$ C/N
$d_{51}=d_{42}$	–	5.84	$10^{-10}$ C/N

**Table 2:** Piezoelectric-based microdevice geometric specifications.

Label	Value (μm)	Label	Value (μm)
$L_{MeP}$	300	$h_{M}$	10
$L_{g}$	270	$h_{C}$	36
$d_{i}$	100	$h_{g}$	119
$d_{e}$	500	$s_{g}$	120
Out-of-plane thickness			10

**Table 3:** Voltage functions $V_{x}\left(t\right)$ and $V_{y}\left(t\right)$ required to generate the desired pathways.

Trajectory			Voltage Functions	
Label	$f_{x}\left(t\right)$	$f_{y}\left(t\right)$	$V_{x}\left(t\right)$	$V_{y}\left(t\right)$
Circular	acos(t)	asin(t)	40cos(t)	5.473sin(t)
Elliptical	acos(t)	bsin(t)	40cos(t)	2sin(t)
Straight line	acos(t)	b(t)	40cos(t)	5.473sin(t)
Spiral	$a\left(e^{\frac{t}{10}}-1\right)\cos\left(5t\right)$	$b\left(e^{\frac{t}{10}}-1\right)\sin\left(5t\right)$	$40\left(e^{\frac{t}{10}}-1\right)\cos\left(5t\right)$	$5.473\left(e^{\frac{t}{10}}-1\right)\sin\left(5t\right)$
Cycloidal	$\frac{a}{2\pi }\left(t+\sin\left(10t\right)\right)$	bcos(10t)	$\frac{40}{2\pi }\left(t+\sin\left(10t\right)\right)$	3cos(10t)
Infinity	asin(t)	bsin(2t)	40sin(t)	5.473sin(2t)
Star	asin(12t)cos(t)	bsin(12t)sin(t)	40sin(12t)cos(t)	5.473sin(12t)sin(t)
Cardioid	a(2∗cos(t)+cos(2t))	b∗(2∗sin(t)+sin(2t))	15(2∗cos(t)+cos(2t))	2∗(2∗sin(t)+sin(2t))
Nephroid	a(4∗cos(t)+cos(4t))	b∗(4∗sin(t)+sin(4t))	7.5(4∗cos(t)+cos(4t))	1∗(4∗sin(t)+sin(4t))
Four-leaf clover	asin(2t)cos(t)	bsin(2t)sin(t)	40sin(2t)cos(t)	5.473sin(2t)sin(t)
Lissajous 1	asin(2t)	bsin(3t)	40sin(2t)	5.473sin(3t)
Lissajous 2	asin(10t)	bsin(7t)	40sin(10t)	5.473sin(7t)

## Nomenclature

*b*	out of plane thickness
$E_{M}$	Young’s modulus of silicon
$E_{p}$	Young’s modulus of PZT
$h_{M}$	thickness of silicon
$h_{P}$	thickness of PZT
$M_{a}$	bending moment applied by PZTs
*u*	displacement of the specimen along the *x*-axis
*v*	displacement of the specimen along the *y*-axis
$V_{x},V_{y}$	electrical voltages supplied to PZTs
$x_{p}\left(t\right),y_{p}\left(t\right)$	parametric equations of the wanted trajectory
